# Draft Genome Sequence of *Rhodococcus* sp. Strain NCIMB 12038, a Naphthalene-Degrading Bacterium

**DOI:** 10.1128/genomeA.01420-17

**Published:** 2018-01-04

**Authors:** Shamsudeen U. Dandare, Timofey Skvortsov, Ksenia Arkhipova, Christopher C. R. Allen

**Affiliations:** a School of Biological Sciences, Queen’s University Belfast, Belfast, United Kingdom

## Abstract

We report here the draft genome sequence of *Rhodococcus* sp. strain NCIMB 12038, an industrially important bacterium, possessing a large and diverse repertoire of genes involved in the biotransformation of various organic compounds, including naphthalene.

## GENOME ANNOUNCEMENT

The members of the genus *Rhodococcus* are genetically and metabolically diverse, which allows them to adapt to a wide range of environmental conditions and utilize various organic compounds as energy and nutrient sources, including highly toxic ones. *Rhodococcus* sp. strain NCIMB 12038, originally isolated by Larkin and Day from garden soil in 1983, accepts carbaryl as a sole carbon and nitrogen source (1, 2). The ability of this strain to metabolize and degrade complex organic molecules, including naphthalene, and produce valuable activated aromatic compounds, as well as its involvement in fossil fuel biodesulfurization, makes it an industrially and ecologically important microorganism (3, 4).

*Rhodococcus* sp. strain NCIMB 12038 was revived from a freeze-dried sample (prepared ca. 1998 in our laboratory) by plating on LB agar; a single colony was then picked and grown on solid minimal salt medium (MSM) with the addition of naphthalene as the only carbon source. Subsequently, a single colony was picked and grown in liquid MSM with the direct addition of 2 g/liter of naphthalene to the medium. In all growth experiments, bacteria were grown at 25°C to late exponential phase.

Cell cultures were pelleted, and genomic DNA was extracted using the FastDNA SPIN kit for soil (MPBio, Solon, OH, USA) following the manufacturer’s protocol. Whole-genome sequencing was performed at the MR DNA sequencing facility (Shallowater, TX, USA) using the Illumina MiSeq platform. The paired-end 150-bp sequence reads generated were assembled at MR DNA using NGen DNA assembly software (DNAStar, Inc., Madison, WI, USA), producing 112 contigs, with an average coverage of 40×. Minimus2 (5) was used to merge contigs with overlaps, and the resulting set of 109 contigs was analyzed with CheckM (6), which characterized the draft genome as 99.5% complete. The assembly was annotated online using the NCBI Prokaryotic Genome Annotation Pipeline (PGAP) (7).

The draft genome of *Rhodococcus* sp. NCIMB 12038 is 9.3 Mb long and has a GC content of 67.2%, which is in the range of values characteristic to other members of the genus (8). A total of 8,506 genes were predicted by NCBI PGAP, including 8,121 protein-coding sequences, 321 pseudogenes, and 64 RNA genes. Among the identified RNA genes, 49 were annotated as tRNAs and 3 as noncoding RNAs, while 4 copies of 5S, 3 copies of 16S, and 5 copies of 23S rRNAs were detected in the draft genome. SpecI (9) prediction and comparative analysis of the 16S rRNA sequences identified *R. opacus* as the closest related species. Annotation of the protein-coding genes with RASTtk (10) assigned 26% of them to functional categories of SEED subsystems, while analysis with AntiSmash version 4.0 (11) predicted 160 biosynthetic clusters. IslandViewer (12) predicted 76 genomic islands; several putative insertion sequences and prophages were also detected. No clustered regularly interspaced short palindromic repeat (CRISPR) arrays were found after an inspection with CRISPRDetect (13).

### Accession number(s).

This whole-genome shotgun project has been deposited at DDBJ/ENA/GenBank under the accession number NHML00000000. The version described in this paper is the first version, NHML01000000.
